# High prevalence of carbapenem-resistant *Enterobacter cloacae* complex in a tertiary hospital over a decade

**DOI:** 10.1128/spectrum.00780-24

**Published:** 2024-10-30

**Authors:** Shiqi Cai, Jingjing Quan, Zhengan Wang, Huangdu Hu, Xinhong Han, Yan Jiang, Qing Yang, Yunsong Yu, Zhihui Zhou

**Affiliations:** 1 Department of Infectious Diseases, Sir Run Run Shaw Hospital, Zhejiang University School of Medicine, Hangzhou, China; 2 Key laboratory of Microbial Technology and Bioinformatics of Zhejiang Province, Hangzhou, China; 3 Regional Medical Center for National Institute of Respiratory Diseases, Sir Run Run Shaw Hospital, Zhejiang University School of Medicine, Hangzhou, China; 4 Department of Laboratory Medicine, The First Affiliated Hospital, Zhejiang University School of Medicine, Hangzhou, China; 5 State Key Laboratory for Diagnosis and Treatment of Infectious Diseases, National Clinical Research Center for Infectious Diseases, Collaborative Innovation Center for Diagnosis and Treatment of Infectious Diseases, The First Affiliated Hospital, Zhejiang University School of Medicine, Hangzhou, China; JMI Laboratories, North Liberty, Iowa, USA

**Keywords:** *Enterobacter cloacae *complex, carbapenem resistance, carbapenemase-producing *Enterobacteriaceae*, molecular epidemiology

## Abstract

The aim of this study was to explore the mechanisms and molecular epidemiology of carbapenem resistance in the carbapenem-resistant *Enterobacter cloacae* complex (CRECC) over a decade in a tertiary hospital in Zhejiang, China. From January 2011 to December 2021, we collected a total of 931 *Enterobacter cloacae* complex (ECC) isolates from a tertiary hospital in Zhejiang, China. Antimicrobial susceptibility tests were performed. Whole-genome sequencing was used to analyze the molecular characteristics of the CRECC isolates. For carbapenem-resistant strains, efflux inhibitor assay and quantitative real-time PCR (qRT-PCR) were performed to evaluate the function of efflux pumps. A total of 82 CRECC isolates were detected, and the rate of resistance for carbapenems was 8.8%, increasing from 5.5% in 2011 to 18.3% in 2019, with an overall increasing trend, with *Enterobacter hormaechei* subsp*. hoffmannii* being the predominant species. Among the CRECC, 24 (24/931) isolates were found to produce carbapenemases, including NDM-1, NDM-5, IMP-4, and KPC-2. Among all carbapenemases, NDM-1 was the most prevalent, accounting for 62.5% (15/24) of carbapenemases, followed by NDM-5 (5/24). Genes encoding extended-spectrum beta-lactamases (47/82) and AmpC (76/82) were also identified, with *bla*_SHV-12_ and *bla*_ACT_ being the predominant ones, respectively. Multilocus sequence typing revealed 28 different sequence types, among which ST78 was the predominant, followed by ST93 and ST177. IncFIB was the most common type of plasmid replicon. Efflux inhibitor assay and qRT-PCR indicated that the overexpression of efflux pumps was involved in carbapenem resistance mechanisms. Additionally, disrupted outer membrane proteins also contribute to carbapenem resistance. The detection rate of CRECC was rising in the tertiary hospital. *Bla*_NDM-1_ and *bla*_NDM-5_ were the main carbapenem resistance genes. Our study revealed the presence of carbapenem-resistant ECC strains, emphasizing the need for effective infection prevention approaches to reduce the prevalence of CRECC.

**IMPORTANCE:**

The emergence and spread of the carbapenem-resistant *Enterobacter cloacae* complex (CRECC) have become a significant public health problem. CRECC strains frequently harbor multiple drug resistance genes and can be epidemic within healthcare facilities. The study explored the characteristics and prevalence of CRECC strains in the same hospital over a decade, which provides a theoretical basis for epidemiologic surveillance and clinical treatment.

## INTRODUCTION

The *Enterobacter cloacae* complex (ECC) is composed of a group of Gram-negative bacteria belonging to the genus Enterobacter in the family *Enterobacteriaceae*. The ECC is a common opportunistic pathogen, which is responsibility to a series of hospital-acquired infections, including endocarditis, pneumonia, and septic arthritis (1). And urinary tract infection, respiratory infections, and skin and soft-tissue infections are the most common hospital-associated *Enterobacter* infections (2). The following species are included: *Enterobacter asburiae*, *Enterobacter carcinogenus*, *Enterobacter cloacae*, *Enterobacter hormaechei*, *Enterobacter kobei*, *Enterobacter nimipressuralis*, and *Enterobacter mori* (3). Worldwide, with the spread of extended-spectrum beta-lactamases (ESBLs) and carbapenemases, ECC has become the third most common Enterobacteriaceae bacterium involved in hospital-acquired infections, behind *Escherichia coli* and *Klebsiella pneumoniae* (4).

Carbapenems have been considered a last-resort treatment for multidrug-resistant Gram-negative bacilli isolates. Nevertheless, according to CHINET, a comprehensive antimicrobial resistance surveillance network in China, there has been a steady increase in the rates of resistance to meropenem and imipenem among *Enterobacteriaceae* spp. Carbapenem-resistant Enterobacteriaceae complex (CRECC) is increasingly observed over world (5, 6) and frequently resistant to a variety of antibiotics, posing a great challenge to clinical treatment.

The present studies suggest that the major carbapenem resistance mechanism of ECC involves the production of carbapenemases, and the prevalent carbapenemases include KPC, NDM, IMP, and VIM (7, 8), with KPC predominating (9). In China, however, the most common carbapenemase is NDM-1 (10–12). New Delhi metallo-beta-lactamases 1 (NDM-1) is Ambler class B Metallo-β-lactamases (MBLs) that is capable of conferring resistance to all β-lactams except aztreonam (13). This gene was first reported in 2009 in *K. pneumoniae* (14) and subsequently found in clinical strains in several countries. In China, *bla*_NDM-1_ was detected in various places including Chongqing, and Henan. In addition, it was reported that NDM-1 and NDM-5 were the main carbapenemases in a hospital in northeast China (15).

The production of ESBLs or AmpC and the overexpression of efflux pumps, combined with decreased expression of outer membrane proteins (OMPs), also contribute to carbapenem resistance (16, 17). The main types of ESBLs found in *Enterobacter* spp. are SHV, TEM, and CTX-M (18, 19). Notably, among these ESBLs, *E. cloacae* complex positivity for CTX-M is the most common (20). Drug-resistant genes can be transmitted through multiple mobile elements such as plasmids, transposons, and/or insertion sequences (21) and have been identified in different geographical regions. To date, the involvement of plasmids of incompatibility group X type 3 (IncX3) has been reported with increasing frequency in multiple carbapenem-resistant *Enterobacteriaceae*. IncX3 plasmids are a common plasmid type associated with *bla*_carbapenemase_, leading to the international transfer of carbapenem gene dissemination (22). And IncX3 plasmid harboring *bla*_NDM_ is deemed as the primary vehicle of *bla*_NDM_ transmission (23, 24). Whereas KPC is traditionally associated with IncF-type plasmids. Previous studies have identified considerable sequence types (STs) and substantial clonal diversity, as well as the existence of several potential high-risk clones (25, 26). Nevertheless, long-term consecutive investigations of the CRECC are still uncommon in China.

The present study was conducted to explore the prevalence and mechanisms of carbapenem resistance in the ECC over consecutive years in a tertiary care hospital in China.

## MATERIALS AND METHODS

### Bacterial isolates (sample collection) and strain information

From January 2011 to December 2021, we collected a total of 931 non-repetitive ECC isolates from Ningbo Medical Center Lihuili Hospital in Zhejiang, China. All the isolates were identified by VITEK 2 system (bioMérieux, Marcy-l’Étoile, France).

### Antimicrobial susceptibility test

The isolates we collected were cultured on Mueller-Hinton agar (MHA). Minimum inhibitory concentrations of meropenem (MEM), imipenem (IPM), and ertapenem (ETP) were performed using the agar dilution method according to CLSI guidelines (M100-Ed33). The *E. coli* ATCC 25922 was regarded as the quality control, and the results were interpreted according to the Clinical and Laboratory Standards Institute (CLSI) 2023 guidelines (M100-Ed33).

### Whole-genome sequencing and data analysis

The genomic DNA was extracted by using a QIAamp DNA Mini Kit (Qiagen, Hilden, Germany) and subjected to whole-genome sequencing on the Illumina HiSeq × Ten platform (Illumina, San Diego, USA) with a 150-bp paired-end strategy at Zhejiang Weishu (Hangzhou, China). The raw sequencing reads were assembled into contigs using shovill (https://github.com/tseemann/shovill), and unqualified contigs were eliminated (coverage < 10 or length < 200 bp). A total of 82 CRECC strains with second-generation genome data were eventually obtained.

Multilocus sequence typing (MLST) was based on the Center for Genomic Epidemiology guidelines (https://github.com/tseemann/mlst). Seven housekeeping genes (*dnaA*, *fusA*, *gyrB*, *leuS, pyrG*, *rplB*, and *rpoB*) were regarded as the MLST database.

### Molecular characteristic analysis and detection of antimicrobial resistance genes

Specific identification was performed using JSpeciesWS based on the whole genome sequences (http://jspecies.ribohost.com/jspeciesws/). Antibiotic-resistant genes and plasmid replicons were analyzed using ABRicate v0.8.13 (https://github.com/tseemann/abricate) with the NCBI, AMRFinderPlus, and PlasmidFinder databases, respectively. Prokka v1.14.6 (https://github.com/tseemann/prokka) was used for open reading frame (ORF) annotation. Insertion sequences were determined using ISFinder (https://isfinder.biotoul.fr/). Virulence genes were identified using Pathogenwatch (https://pathogen.watch). To determine carbapenemase production, the modified carbapenem inactivation method (mCIM) and the EDTA-modified carbapenem inactivation method (eCIM) were utilized. The core genome alignment file was achieved with the panaroo pipeline (version 1.2.7) in strict mode (27). The maximum-likelihood phylogenetic tree was generated using the IQ-TREE v2.1.2 (28). The phylogenetic tree was visualized using iTOL v6 (https://itol.embl.de/). The Single nucleotide polymorphism (SNP) number of each isolate was calculated using Snp-dists (https://github.com/tseemann/snp-dists).

Comparison of *ompC*, *ompF*, and *ompX* gene sequences, using the sequences of standard stain obtained from the NCBI (https://www.ncbi.nlm.nih.gov) database under accession number NZ_JAMQCM010000001 as a reference. The strains were considered to have inactivation of the membrane pore protein gene when there was a large insertion, premature termination, or deletion of a large segment of the gene.

### Efflux inhibitor assay

In order to detect whether the efflux pump has an effect on drug resistance, MICs of ertapenem were determined in the presence or absence of an efflux pump inhibitor: carbonyl cyanide-m-chloro phenylhydrazone (CCCP) (8 µg/mL). A series of pre-tests with concentration gradient reagents were conducted to determine that this concentration of reagent will not affect the growth and survival of the strains. This concentration of reagent will not affect the growth and survival of the strain. CCCP solutions use dimethyl sulfoxide (DMSO) as the solvent and then Mueller-Hinton agar (MHA) plates containing a series of twofold-concentration increments of imipenem, meropenem, and ertapenem in conjunction with CCCP (8 µg/mL). For the same strain, the MIC value of the group without CCCP was used as a reference. When the efflux pump inhibitors were present, a reduction of at least fourfold in the MICs was considered indicative of efflux. The plates were incubated for 18 to 24 h at 37°C, and then, the values were read.

### Quantitative real-time PCR for the expression of the efflux pump

The RNAs of three CRECC strains were extracted using the Bacterial RNA Miniprep Kit (Biomiga, Shanghai, China). cDNA was synthesized from 500 ng of total RNA with the PrimeScript RT Reagent Kit (TaKaRa, Shiga, Japan) according to the manufacturer’s instructions. quantitative real-time Polymerase Chain Reaction (qRT-PCR) primer sequences are shown in Table S1. The expression levels of the efflux pump genes (*acrA*, *acrB*, and *tolC*) were performed by quantitative real-time PCR. The internal gene was chosen to be the *rpoB* gene, and the standard strain *Enterobacter cloacae* ATCC 700323 was used as control isolate. Quantitative real-time PCR was performed using SYBR Green Master Mix (TaKaRa) and an ABI Prism System (Applied Biosystems, Carlsbad, CA). The comparative threshold cycle 2-Ct technique was used to examine the target gene quantification. All experiments were carried out three times, once in duplicate and once independently.

## RESULTS

### Characteristics of the collected strain samples

**Fig 1:**
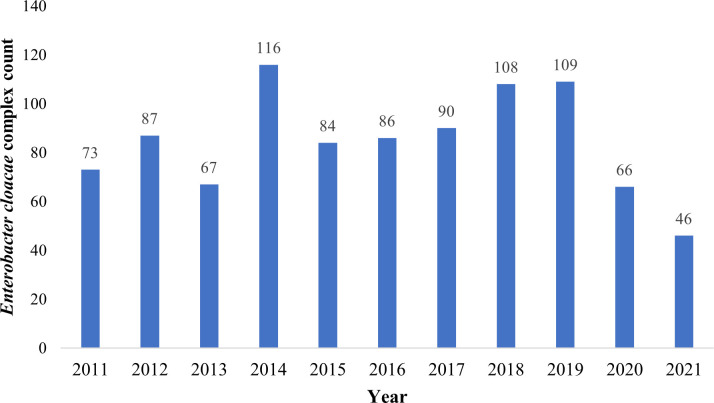
Number of *Enterobacter cloacae* complex over 11 consecutive years.

Strain isolates were collected for 11 consecutive years from 2011 to 2021 (Fig. 1).

The strain isolates were collected from various anatomical sites, most of which were from the respiratory tract, including sputum and bronchial lavage (*n* = 532, 57.1%). After the respiratory tract, the most common primary sites of isolation were the urinary tract (*n* = 102, 11.0%), digestive tract (*n* = 88, 9.5%), etc. In different years, respiratory specimens were nearly always predominant.

The strains were submitted by more than 20 different departments of the hospital, with the main department being the following: the Department of Neurosurgery (*n* = 157, 16.9%), the Department of General Surgery (*n* = 122, 13.1%), the Department of Respiratory Medicine (*n* = 108, 11.6%), etc. The remaining 16 isolates were of unknown origin section. Notably, neurosurgery was the top department, followed closely by general surgery. Detailed information is provided in the supplemental material.

### Antimicrobial susceptibility test

**Fig 2:**
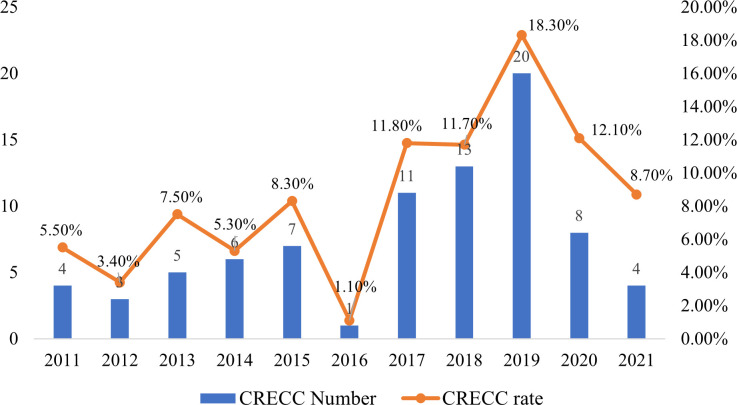
Trends in the number and detection rate of CRECC isolates from 2011–2021.

The 931 isolates were determined with three carbapenems including imipenem, meropenem, and ertapenem. The total number of carbapenem-resistant ECC isolates was 82 (8.6%), and these isolates were resistant to at least one carbapenem. The results revealed a relatively rapid upward trend in the CRECC detection rate over the past 11 years, from 5.5% in 2011 to 18.3% in 2019 (Fig. 2). However, the detection rate exhibited a downward trend in 2020 and 2021. Nevertheless, the lowest rate of detection in 2016 was only 1.1%, which would potentially be attributed to sampling error.

**Fig 3:**
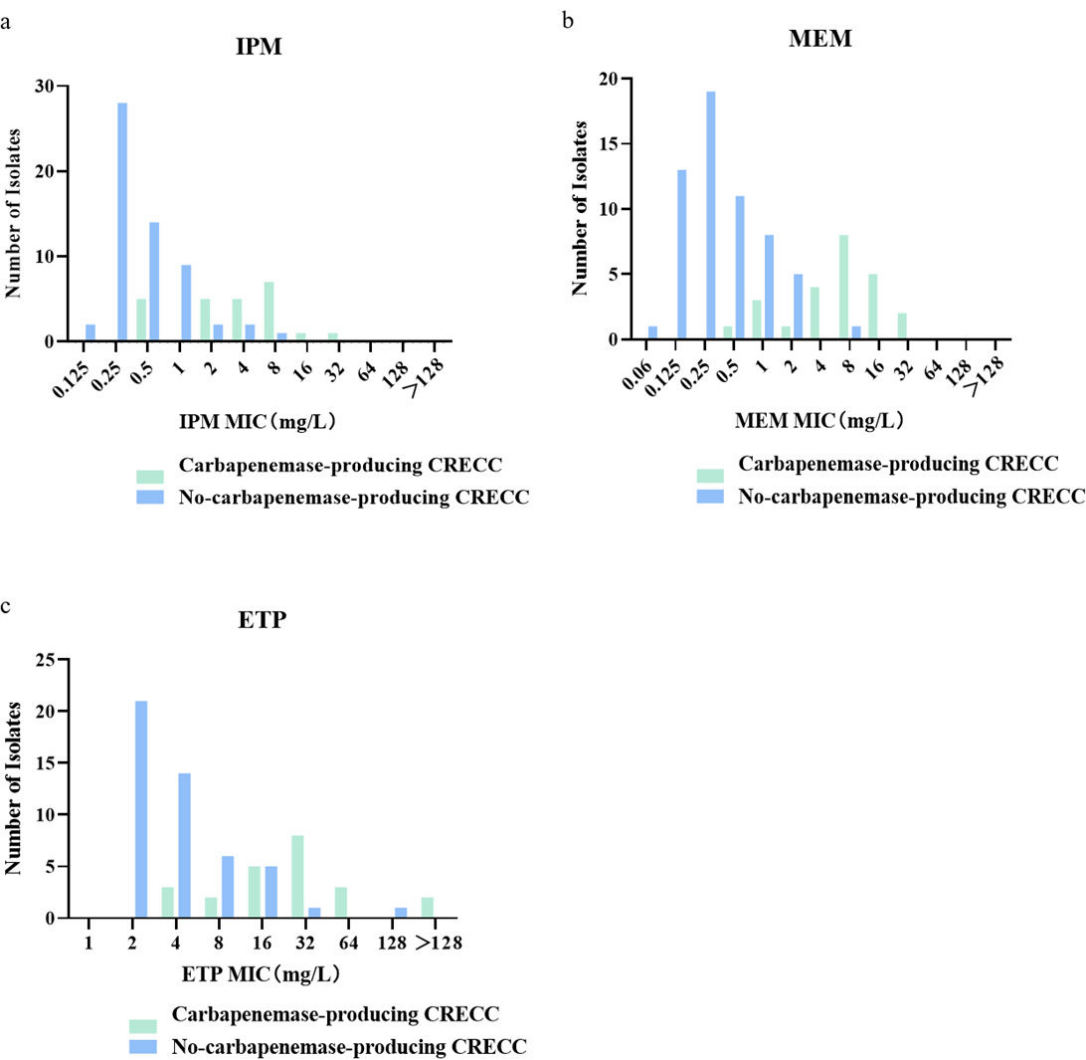
MIC distributions of three carbapenem drugs. (a) MIC distribution of imipenem; (b) MIC distribution of meropenem; (c) MIC distribution of ertapenem.

Resistance rates also varied among the three carbapenems, with ertapenem having the highest rate of resistance (8.8%, 82/931). The overall rates of resistance for meropenem and imipenem were 2.1% (20/931) and 1.8% (17/931), respectively. The rates of resistance for each of the three carbapenem drugs in each of the 11 consecutive years are shown in Fig. S1 in the supplemental material. The rate of resistance to ertapenem was significantly greater than that to the other two drugs, and the overall trend was to increase annually. Among the CRECC strains, the MIC for ertapenem ranged from 2 µg/mL to >128 µg/mL. However, the MIC range for imipenem was 0.125 to 32 mg/L, while the MIC range for meropenem was 0.06 to 32 mg/L. The MIC ranges of the three carbapenem drugs were significantly greater for the carbapenemase-producing strains than for the non-carbapenemase-producing strains (Fig. 3). Among the strains resistant to meropenem and ertapenem, the percentages of carbapenemase-producing CRECC were 95% and 82.4%, respectively.

In terms of evaluating the characteristics of the carbapenem-resistant ECC isolates, the main departments are as follows: respiratory medicine (*n* = 12, 14.6%), neurosurgery (*n* = 12, 14.6%), and general surgery (*n* = 10, 12.2%). The numbers of isolates from other departments were in single digits.

Regarding the species of the CRECC isolates, the most prevalent species discovered was *E. hormaechei* subsp. *hoffmannii* (31, 31/82), followed by *E. hormaechei* subsp. *steigerwaltii* (16, 19.5%), *E. hormaechei* subsp. *xiangfangensis* (14, 17.1%), *E. hormaechei* subsp. *oharae* (2, 2.4%), *Enterobacter roggenkampii* (7, 8.5%), *E. kobei* (6, 7.3%), *E. asburiae* (3, 4.9%), *E. cloacae* (2, 2.4%), and *Enterobacter ludwigii* (1, 1.2%). (Table 1)

**TABLE 1:** Species identified in carbapenem-resistant *Enterobacter cloacae* complex isolates

Species		Number	Proportion
*E. hormaechei* subsp.	*E. hormaechei* subsp. *hoffmannii*	31	37.8%
	*E. hormaechei* subsp. *steigerwaltii*	16	19.5%
	*E. hormaechei* subsp. *xiangfangensis*	14	17.1%
	*E. hormaechei* subsp. *oharae*	2	2.4%
*E. roggenkampii*		7	8.5%
*E. kobei*		6	7.3%
*E. asburiae*		3	3.%
*E. cloacae*		2	2.4%
*E. ludwigii*		1	1.2%

### Multilocus sequence typing and phylogenetic tree

**Fig 4:**
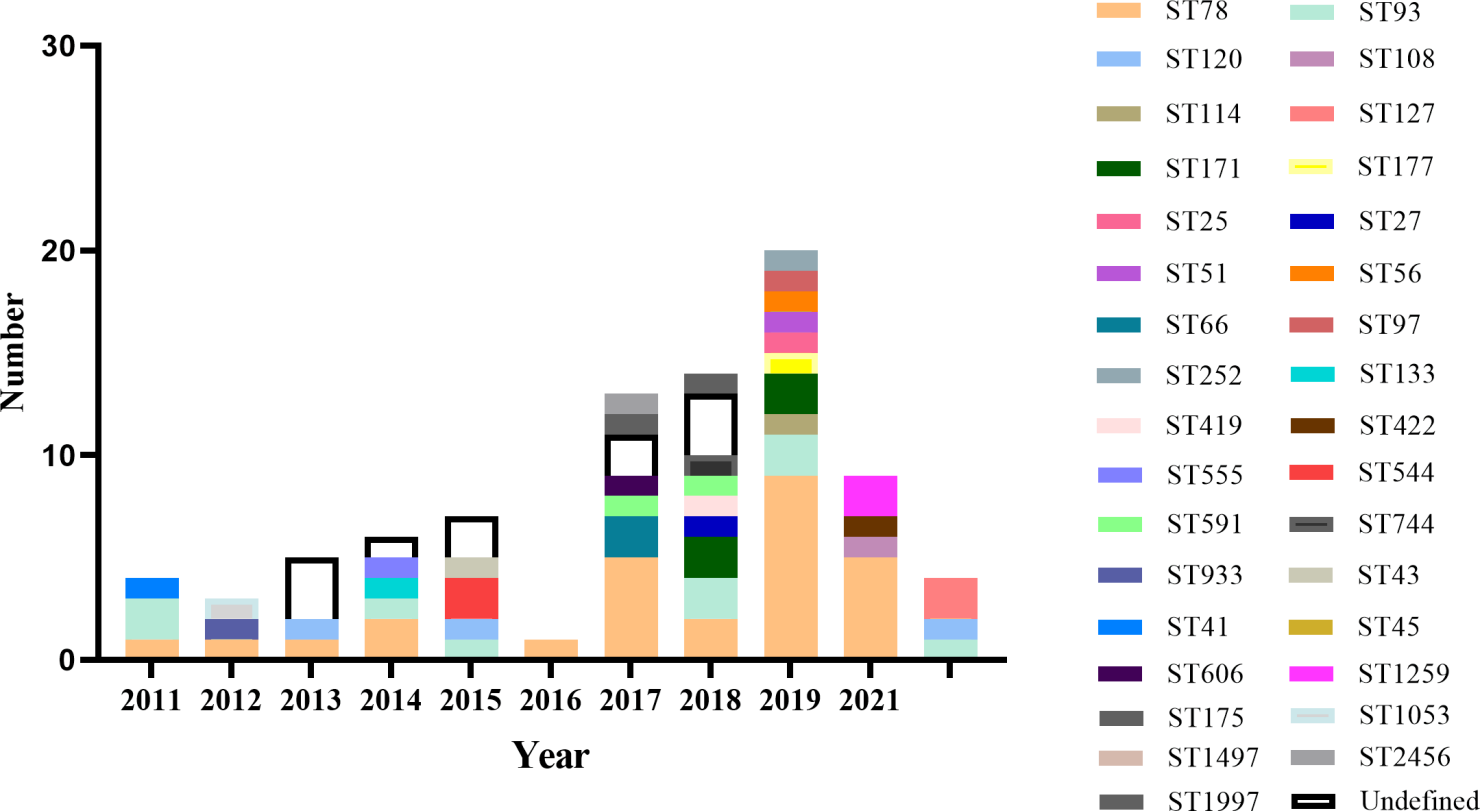
ST type distribution over each year.

The MLST results revealed 28 different STs, among which ST78 was the predominant ST type, accounting for 32.9% (*n* = 27), followed by ST93, ST120, and ST171. However, of the 28 STs found, 12 strains are novel STs. We found that ST types are diverse and dispersed. We also investigated the relationship between different years and ST types (Fig. 4). Also, the number and distribution of sequence types varied in different years, with ST types being most abundant in 2019. Although ST78 was the most dominant ST type, it was not present in 2015 and 2021. The predominant ST type of *E. hormaechei* subsp. *hoffmannii* is also ST78. As for ST78, strains are significantly more prevalent in 2019, suggesting a potential ST78 outbreak epidemic in that year.

**Fig 5:**
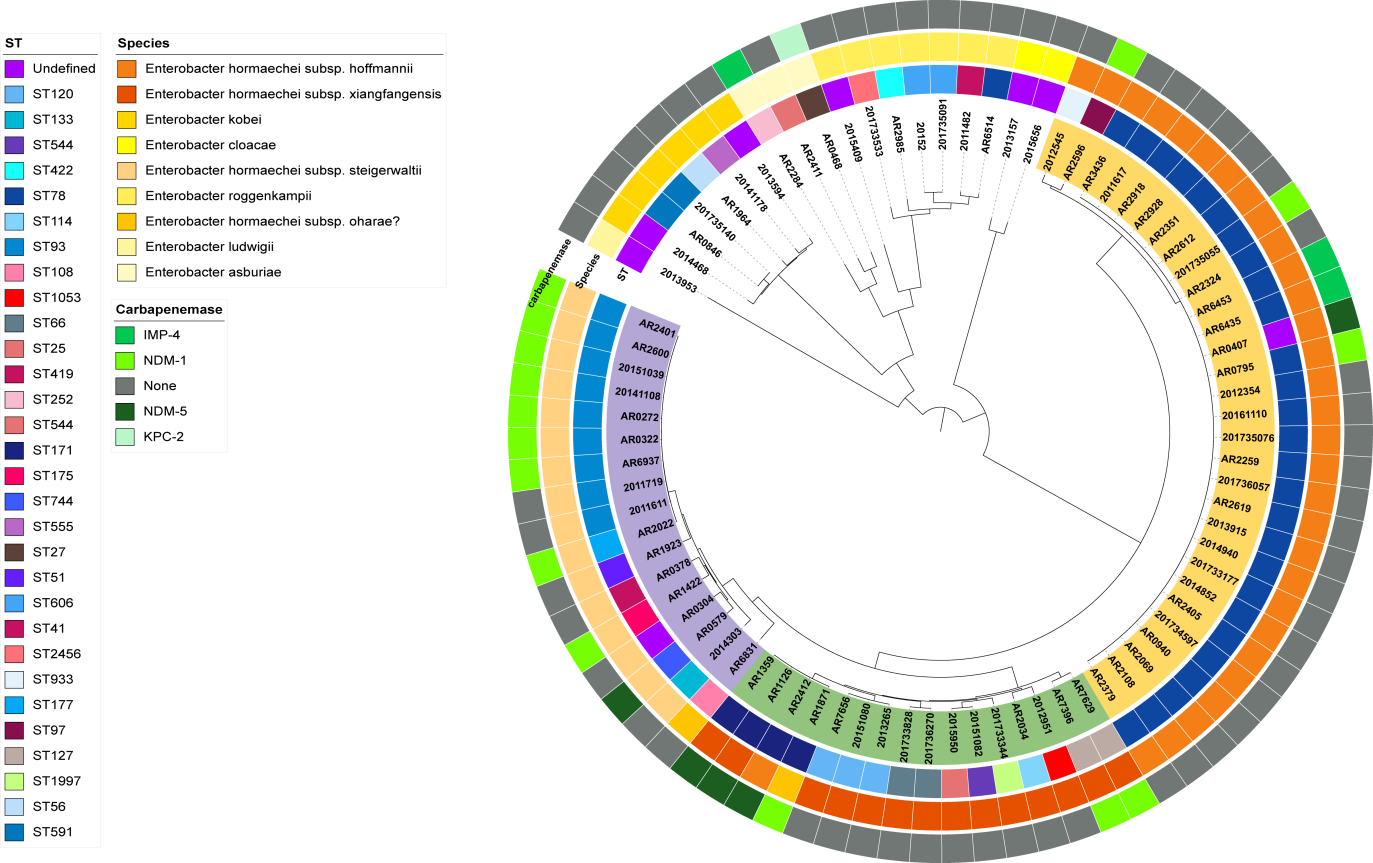
Phylogenetic analysis of 82 CRECC isolates. The isolates were divided into four clusters, which were in accordance with the species. From inside to outside, the sequence types (STs), species of the ECCs, and genes encoding carbapenemases for all *E. cloacae* strains are successively listed in each layer.

Phylogenetic tree was constructed by the maximum-likelihood method reflecting the genetic relationships among all 82 CRECC isolates in the study. The isolates were divided into four clusters, which were in accordance with the species. From inside to outside, the sequence types (STs), species of the ECCs, and genes encoding carbapenemases for all *E. cloacae* strains are successively listed in each layer (Fig. 5).

**Fig 6:**
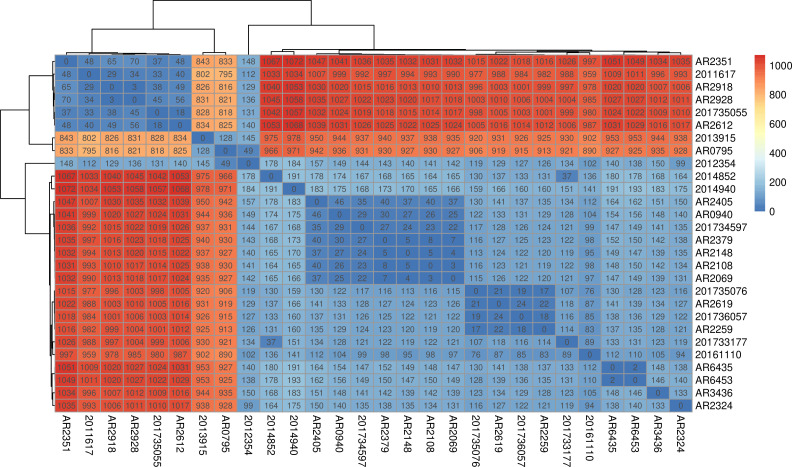
SNP analysis of CRECC of ST78.

We further analyzed the core genome SNP homology of CRECC of ST78 (Fig. 6), and in general, most of the strains had large SNP differences, and only a few strains had small SNP differences, and there is no obvious clonal transmission of ST78 isolates considered.

### Detection of genes encoding carbapenemases, AmpC, and ESBLs in CRECC isolates

In addition, this study detected antimicrobial resistance genes of CRECC. The genes encoding carbapenemase, ESBL, and AmpC were analyzed. First, there were 24 carbapenemase-producing ECC (29.3%, 24/82), producing a total of eight types of carbapenemases, which were NMD-1 (*n* = 15), NDM-5 (*n* = 5), IMP-4 (*n* = 3), and KPC-2 (*n* = 1). We also performed the mCIM test at the same time, and both results were positive. About the types of carbapenemase, NDM-1 was the most common (15/24), with 60% (9 isolates) belonging to *E. hormaechei* subsp. *steigerwaltii*, and the predominant ST type was ST93. Among NDM-5-positive CRECC isolates, 64.6% (53/82) belonged to *E. hormaechei* subsp. *hoffmannii*. All strains were positive for mCIM and eCIM tests. Also, plasmid types of carbapenemase-producing strains were analyzed, with IncX3_1 type plasmid being most common. Information on all the carbapenemase-producing *E. cloacae* isolates is given in the Table 2.

**TABLE 2:** Molecular characteristics of the carbapenemase-producing *E. cloacae* isolates^*a*^

Year	Isolates	MLST	Specimen	Carbapenemase	Specials	mCIM/eCIM	ESBLs	Inc type	Assembled contigs	Insertion sequences
2014	1108	ST93	Sputum	NDM-1	*E. hormaechei* subsp. *steigerwaltii*	+	SHV-12	IncX3	contig00049	IS5, ISAba125, IS3000, ISKpn26
2015	1039	ST93	Sputum	NDM-1	*E. hormaechei* subsp. *steigerwaltii*	+	SHV-12	IncX3	contig00044	IS5, ISAba125, IS3000, ISKpn26
2017	35055	ST78	Sputum	NDM-1	*E. hormaechei* subsp. *hoffmannii*	+	CTX-M-3	IncFIB	contig00038	IS3, IS5
2018	AR0272	ST93	Urine	NDM-1	*E. hormaechei* subsp. *steigerwaltii*	+	SHV-12	IncX3	contig00044	IS5, ISAba125, IS3000, ISKpn26
2018	AR0322	ST93	urine	NDM-1	*E. hormaechei* subsp. *steigerwaltii*	+	SHV-12	IncX3	contig00044	IS5, ISAba125, IS3000, ISKpn26
2018	AR0407	/	Sputum	NDM-5	*E. hormaechei* subsp. *hoffmannii*	+	CTX-M-3	IncX3/IncFII/IncHI2/IncHI2A/IncFIB	contig00045	/
2018	AR0468	ST27	Sputum	KPC-2	*E. asburiae*	+	TEM-150	IncFII/IncX5	contig00033	IS481, IS1182
2018	AR0579	ST744	Drainage fluid	NDM-5	*E. hormaechei* subsp. *steigerwaltii*	+	CTX-M-9 SHV-12	IncHI2/IncHI2A/IncX3	contig00028	IS630
2018	AR0795	ST78	Sputum	NDM-1	*E. hormaechei* subsp. *hoffmannii*	+	SHV-12	IncFIB/IncX3	contig00023	IS5, ISAba125, IS3000, ISKpn26
2018	AR1126	ST171	Sputum	NDM-5	*E. hormaechei* subsp. *xiangfangensis*	+	CTX-M-65	IncX3/IncFIB/IncFIA/IncR	contig00030	IS5, ISAba125, IS3000, ISKpn26
2018	AR1359	ST171	Sputum	NDM-5	*E. hormaechei* subsp. *xiangfangensis*	+	CTX-M-65	IncX3/IncFIB/IncFIA/IncR	contig00032	IS5, ISAba125, ISKpn26
2018	AR1422	/	Sputum	NDM-1	*E. hormaechei* subsp. *steigerwaltii*	+	SHV-12	IncX3/IncHI2/IncHI2A	contig00045	/
2019	AR1871	ST177	Sputum	NDM-1	*E. hormaechei* subsp. *oharae*	+	SHV-12	IncX3/IncFIB	contig00044	/
2019	AR2022	ST177	Sputum	NDM-1	*E. hormaechei* subsp. *steigerwaltii*	+	SHV-12	IncX3	contig00018	IS5, ISAba125, IS3000, ISKpn26, ISKox3, ISCfr12, ISEc53
2019	AR2284	ST252	Sputum	IMP-4	*E. asburiae*	+	CTX-M-9	IncHI2A/IncFIB/IncFII	contig00066	/
2019	AR2401	ST93	Sputum	NDM-1	*E. hormaechei* subsp. *steigerwaltii*	+	SHV-12	IncX3	contig00047	IS5, ISAba125, IS3000, ISKpn26
2019	AR2412	ST171	Sputum	NDM-5	*E. hormaechei* subsp. *hoffmannii*	+	CTX-M-65	IncFII/ IncHI2A/IncHI2/IncN	contig00029	IS5, ISAba125, ISKpn26
2019	AR2596	ST97	Urine	NDM-1	*E. hormaechei* subsp. *hoffmannii*	+	CTX-M-9	IncFII/IncHI2/IncN/IncHI2A	contig00054	ISKpn19, ISEc51
2019	AR2600	ST93	Drainage fluid	NDM-1	*E. hormaechei* subsp. *steigerwaltii*	+	SHV-12	IncX3	contig00049	IS5, ISAba125, IS3000, ISKpn26
2020	AR6435	ST78	Urine	IMP-4	*E. hormaechei* subsp. *hoffmannii*	+	CTX-M-9, SFO-1	IncFIB/IncHI2A/IncHI2	contig00050	/
2020	AR6453	ST78	Urine	IMP-4	*E. hormaechei* subsp. *hoffmannii*	+	CTX-M-9, SFO-1	IncFIB/IncHI2/IncHI2A	contig00050	/
2021	AR6937	ST93	Leukorrhea	NDM-1	*E. hormaechei* subsp. *steigerwaltii*	+	SHV-12	IncX3	contig00050	IS5, ISAba125, IS3000, ISKpn26
2021	AR7396	ST127	Sputum	NDM-1	*E. hormaechei* subsp. *xiangfangensis*	+	/	IncX3/IncR	contig00020	
2021	AR7629	ST127	Pleural effusion	NDM-1	*E. hormaechei* subsp. *xiangfangensis*	+	/	IncX3/IncR	contig00019	

^*a*^ ESBL, extended-spectrum β-lactamase; ST, sequence type; /,the strains did not harbor *bla*_ESBL_ .

Among the 46 isolates that harbored *bla*_ESBL_, 31 had *bla*_CTX-M_, 22 carried *bla*_SHV-12_, and some isolates harbored multiple ESBLs. As for *bla*_CTX-M_, *bla*_CTX-M-3_ is the most prevalent (*n* = 10, 21.3%), followed by *bla*_CTX-M-9_ (*n* = 9, 19.1%) and *bla*_CTX-M-15_ (*n* = 8, 17.0%) (Table 3). A total of 26 carbapenemase-negative isolates harbored *bla*_ESBL_. Of note, cocarriage of carbapenemase and ESBL was detected in 21 *bla*_ESBL_-positive isolates.

**TABLE 3:** The different types of *bla*_ESBLs_ among carbapenem-resistant *Enterobacter cloacae* complex

Types of *bla*_ESBLs_		Number	Component ratio
*bla*_CTX-M_	*bla*_CTX-M-3_	10	21.7%
	*bla*_CTX-M-9_	9	19.6%
	*bla*_CTX-M-15_	8	17.4%
	*bla*_CTX-M-14_	1	2.2%
	*bla*_CTX-M-65_	3	6.5%
*bla*_SHV_	*bla*_SHV-12_	22	47.8%
*bla*_SFO_	*bla*_SFO-1_	5	10.9%

The AmpC enzymes included CMY, FOX, ACC, LAP, MIR, ACT, MOX, and DHA. All the isolates were positive for AmpC, and genes encoding ACT were the most common, accounting for 92.7% (76/82). In addition, genes encoding LAP, MIR, DHA, and MOX accounted for 7.3% (6/82), 11.0% (9/82), 2.4% (2/82), and 1.2% (1/82). It is worth noting that the carriage of multiple ESBLs and/or AmpC was common (Table 4).

**TABLE 4:** The different types of *bla*_AmpCs_ genes among carbapenem-resistant *Enterobacter cloacae* complex

Types of *bla*_AmpCs_	Number	Component ratio
*bla*_ACT_	76	92.7%
*bla*_MIR_	6	7.3%
*bla*_DHA_	2	2.4%
*bla*_MOX_	1	1.2%

### Types of plasmid replicons in the carbapenem-resistant *Enterobacter cloacae* complex

**Fig 7:**
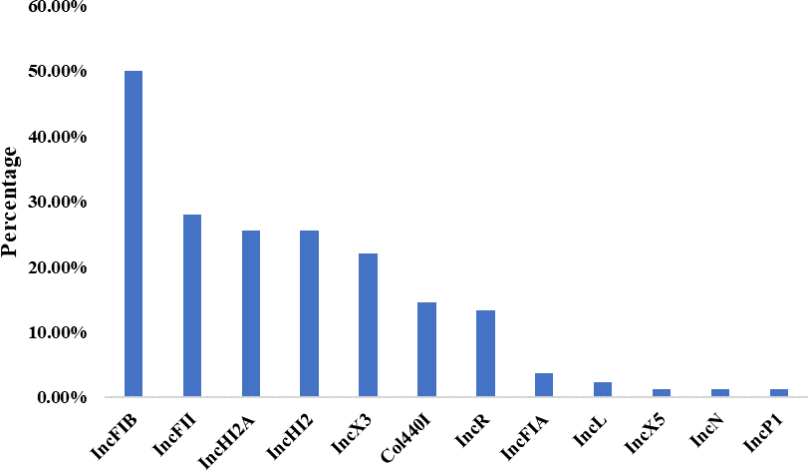
Plasmid replicons of the carbapenem-resistant *Enterobacter cloacae* complex.

In total, different types of plasmid replicons were identified in 85.5% (70/82) of the isolates. IncFIB was the most common accounting for up to 50.6% (41/82), while among carbapenemase-producing stains, IncX3 was the dominant plasmid replicon. The plasmid replicons in the CRECC isolates were shown in Fig. 7.

### Efflux inhibitor assay and expression of genes encoding efflux pump proteins

After the addition of CCCP, the MICs of the three isolates were significantly reduced. We further explored the expression of the efflux pump genes *AcrA*, *AcrB*, and *TolC*. The standard strain used was *Enterobacter cloacae* ATCC 700323. The results showed that, compared with those in ATCC 700323, the expression levels of all the above three genes were increased in the AR846 isolate. The results of efflux pump-related gene expression have been shown in Table S2.

### The deficiency of the outer membrane protein

Among non-carbapenemase-producing strains, we also detected the outer membrane proteins through a comparative analysis of the genomic sequences of *ompC*, *ompF*, and *ompX*. A total of 58 non-carbapenemase-producing isolates exhibiting AmpC enzyme expression possess sequence deletions. Specific results can be found in Table S3. It seems that the deficiency of the outer membrane protein was involved in the carbapenem resistance mechanisms.

## DISCUSSION

As carbapenem becomes more widely used, CRECC are being found more frequently, potentially leading to clinical failure of anti-infection therapy, deserving high attention. In this study, we explored the strain variation of *E. cloacae* in a hospital over 11 consecutive years. In total, 8.8% (82/931) of the carbapenem-resistant ECC isolates was detected. The results revealed that the detection rate of CRECC isolates increased slowly during 2011–2021, with the lowest rates occurring in 2012 and 2016 and the highest rate occurring in 2018. In addition, we found that the most predominant species of CRECC in this hospital was *E. hormaechei* subsp. *hoffmannii*, consistent with previous studies (17). The second most predominant species in this study was *E. hormaechei* subsp. *steigerwaltii*; however, this was the most predominant strain reported in northern China and Japan (15, 29).

In this study, we evaluated resistance profiles, and the total rates of resistance to imipenem and meropenem in the isolated CRECC strains were 2.7% and 1.8%, respectively. The resistance rate to ertapenem, on the other hand, was as high as 8.8%. Some strains were resistant only to ertapenem, the majority of which were non-carbapenemase producing and had a low MIC. The results also implied that various mechanisms may be involved in the resistance of the *E. cloacae* complex to carbapenem antibiotics, with the prevalence of carbapenemase production being non-negligible (30). In addition, the occurrence of ESBLs, overexpression of efflux pumps, excessive synthesis of AmpC, and disrupted membrane permeability were demonstrated to be involved in resistance to carbapenem (17, 30).

A number of scholars worldwide have reported that the *E. cloacae* complex produces carbapenemases, such as NDM-1, KPC-2, IMP-4, and VIM. To date, the occurrence of NDM-1, which was first identified in South Asia, has become increasingly common. Previous research conducted by multiple teams has detected that NDM-1 is the most common carbapenemase in CRECC isolates and focused on its prevalence, impacts on virulence, and other associated traits (31, 32). An outbreak of NDM-1-producing *E. cloacae* was reported from the United Kingdom (33). Notably, in China, *bla*_NDM-1_ has been identified as the dominant drug-resistant pathogen of carbapenemase-producing *Enterobacteriaceae* in both animals and humans (11). Molecular epidemiology studies revealed the prevalence of CRECC strains that produce *bla*_NDM-1_ in Guangdong, Chongqing, Henan (8 of 11), Ningxia (2 of 12), and northwestern China (11, 12, 15, 34, 35). Several research teams in China have discovered an outbreak of the *E. cloacae* complex carrying NDM-1 (36, 37). Considering the prevalence and clonal expansion of NDM-1, NDM-1-producing CRECC isolates should be of great concern. Our investigation revealed that the prevalence of NDM-1 carbapenemase-encoding genes has emerged and spread since 2012, the strains producing NDM-1 also harbored various other resistance genes, including those encoding ESBLs and AmpC enzymes. Furthermore, we identified *bla*_NDM-5_ as an additional significant carbapenemase gene. *Bla*_IMP-4_ has frequently been found in ECC strains, since it was reported in ST74 and ST194 in Korea (38). However, no strains carrying VIM- and OXA-type genes were found in this study.

MLST analysis revealed a wide range of STs and significant genetic diversity among the strains examined. Among the 82 strains analyzed, a total of 28 distinct STs were identified. Notably, ST78 emerged as the predominant ST, followed by ST93, ST171, and ST120, consistent with previous reports of the widespread STs among global. ST78 was common in the majority of years and had the highest number of isolates in 2018, and the predominant strain among those isolates was *E. hormaechei* subsp. *hoffmannii* as well. A variety of investigations have identified ST78 as a high-risk clone that has spread rapidly over the world (39, 40) and is associated with institutional outbreaks (41), deserving active surveillance. An epidemic of the ST78 clone with *bla*_NDM-1_ was identified in a tertiary care hospital in China (36). However, among the carbapenemase-producing isolates, 25% were ST93 with *bla*_NDM-1_. Spread of ST93, a prevalent clone, has been reported in multiple countries including Australia, Belgium, China, Romania, Spain, Thailand, the United States, and Vietnam, expressing carbapenemases such as VIM-1, NDM-1, KPC-2, and OXA-48 (42). A previous epidemiological survey of the ECC revealed that ST120 has also been detected in several locations in China, while ST171 has been identified as a significant CRECC clone with pandemic potential in the United States (39) and has been widely reported in Japan (43). Additionally, a report from Vietnam showed that ST171 was the predominant type of MLST among carbapenemase-producing *E. cloacae* complex strains (44). On the other hand, international high-prevalence clones such as ST66, ST108, and ST114 are less in number in this CRECC, which may be related to institutional and geographic variations. Since the different ST types may have some association with regional specificity. Close monitoring is warranted due to the possibility of drug-resistant epidemics caused by these STs.

Notably, a host of CRECC isolates can acquire mobile genetic elements harboring antimicrobial resistance genes, including plasmids, which may spread horizontally. The dissemination of mobile resistance genes, especially those encoding NDM, may facilitate rapid dissemination of the *bla*_NDM-1_ gene (32). Plasmid analysis revealed 12 types of plasmid replicons, among which IncFIB was the main ones. Compared with other Inc groups, IncF, A/C, and X are the most commonly associated with carbapenemase production. We also found that IncX3 was the main plasmid of isolates harboring *bla*carbapenemase, suggesting an association between *bla* carbapenemase and IncX3. The IncX3 plasmid is crucial for transporting and spreading carbapenemase genes, particularly *bla*_NDM_, and may be one of the most important platforms for *bla*_NDM_ evolution (45, 46). The *bla*_NDM_ is surrounded by a variety of mobile elements in addition to conserved structures, such as the insertion sequence ISAba125, IS3000, IS5, and IS26, which might be involved in the potential dissemination of *bla*_NDM_ (47).

We studied the spread of NDM-1-producing *E. cloacae* ST78 with contribution of IncX3 plasmid. The *bla*_NDM-1_and *bla*_SHV-12_ genes were located on a self-transferable IncX3 plasmid (48). In Japan, ST78 isolates were found to carry *bla*_IMP-1_ on class 1 integrons encoded on multiple plasmids, such as IncHI2, IncW, and IncFIB (49).

Moreover, it exhibits considerable susceptibility toward ECC strains harboring ESBLs or displaying increased expression of AmpC-type cephalosporinase. For most ECC isolates exhibiting low-level resistance to ertapenem and lacking carbapenemase production, the study showed that the combination of AmpC or ESBLs, along with decreased expression of outer membrane proteins, contributes to this phenotype.

The ESBL gene CTX-M was the most popular gene identified in this study. As several previous studies have shown, CTX-M-15 and CTX-M-14 are the most dominant variants in CTX-M enzymes (50). In our results, CTX-M-3 was the dominant variant of the four CTX-M-type ESBLs, which was the most widespread *bla*_CTX-M_ gene and has been observed in Europe, Asia, South America, Africa, and Bulgaria (51). In addition, other ESBLs such as SHV and TEM have also been identified in the *E. cloacae* complex. The results of this study showed that the carriage rate of ESBL genes by CRECC isolates reached 28.6% and most of carbapenemase-producing strains also carried *bla*_ESBLs_. However, ACT type, which has become the most prevalent plasmid-mediated AmpC β-lactamase, has been described in the literature less frequently in *E. cloacae*. Among the remaining 58 strains that did not produce carbapenemase, *bla*_AmpC_ was detected in our study, whereas the prevalence of *bla*_ACT_ may lead to resistance to cephalosporins. Additionally, *bla*_ACT-24_ was the most frequently found gene in the isolates and was found only in *E. hormaechei* subsp. *hoffmannii*.

As shown in previous studies, the susceptibility of ECC isolates to carbapenem can increase when the AcrAB-TolC efflux pump is inhibited. Additionally, CCCP is a pump inhibitor that can compete with antibacterial drugs for binding to bacterial efflux pump proteins (30). The results of the present study suggested that carbapenem resistance may be related to efflux pumps, but they may not play a major role in the mechanism of carbapenem resistance. In AmpC-producing ECC isolates, decreased expression of OmpC and/or OmpF was detected, and this combination may increase the MIC of carbapenem drugs for these strains. A reduction in the permeability of outer membrane proteins was also reported to be involved in carbapenem resistance (16). A study conducted at a hospital in Taiwan demonstrated that the loss of OmpC and OmpF contributed to carbapenem resistance (52).

Studying the characteristics and prevalence of ECC strains in the same hospital over a decade might help detect potential competitive emerging clones. The results may influence clinical decisions regarding appropriate therapies and determine infection control measures.

This study has several limitations. First, although the strains were collected over a decade, the sample size was relatively small and may not fully reflect the molecular epidemiology of CRECC strains. We did not further scrutinize the plasmids with the resistance genes by performing third-generation sequencing. Further investigation into the underlying mechanisms is needed.

### Conclusions

In conclusion, our study indicated that the detection rate of CRECC is rising during 2011–2021 in a tertiary hospital. ST78 was the predominant ST, followed by ST93, ST171, and ST120, and *E. hormaechei* subsp. *hoffmannii* was the most common species. NDM-1 and NDM-5 were the main carbapenemases in CRECC isolates, and CTX-M and SHV were the major ESBLs. Additionally, diversely distinct *bla*_AmpC_ and different replicons were identified, with *bla*_ACT_ and IncFIB being the predominant ones, respectively. The overexpression of efflux pumps and disrupted membrane permeability also contribute to carbapenem resistance. Our analysis showed that it’s quite imperative for illustrating the resistance characteristics of such strains and guiding rational drug use in clinic. It is essential to early screen for carbapenem-resistant pathogens, strengthen surveillance, and report new cases of CRECC infection in healthcare settings.

## SUPPLEMENTAL MATERIAL

Supplemental material spectrum.00780-24-s0001.docx Additional experimental details, Tables S1-S3, and Figure S1.

ASM does not own the copyrights to Supplemental Material that may be linked to, or accessed through, an article. The authors have granted ASM a non-exclusive, world-wide license to publish the Supplemental Material files. Please contact the corresponding author directly for reuse.
