# Supplementary material for: High prevalence of carbapenem-resistant Enterobacter cloacae complex in a tertiary hospital over a decade
Source: Microbiol Spectr. 2024 Oct 30;12(12):e00780-24. doi: 10.1128/spectrum.00780-24 (PMC11619405; doi:10.1128/spectrum.00780-24)
Supplement: Supplemental material — Additional experimental details, Tables S1-S3, and Figure S1. [file spectrum.00780-24-s0001.docx]

**High prevalence of Carbapenem-resistant *Enterobacter cloacae* complex in a tertiary hospital over a decade**

Shiqi Cai^1,2,3^, Jingjing Quan^1,2,3^, Zhengan Wang^1,2,3^, Huangdu Hu^1,2,3^, Xinhong Han^1,2,3^, Yan Jiang^1,2,3^, Qing Yang^4,5^, Yunsong Yu^1,2,3*^, Zhihui Zhou^1,2,3*^

^1^ *Department of Infectious Diseases, Sir Run Run Shaw Hospital, Zhejiang University School of Medicine, Hangzhou, China.*

^2^ *Key laboratory of Microbial Technology and Bioinformatics of Zhejiang Province, Hangzhou, China*

^3^ *Regional Medical Center for National Institute of Respiratory Diseases, Sir Run Run Shaw Hospital, Zhejiang University School of Medicine, Hangzhou, China.*

^4^ *Department of Laboratory Medicine, The First Affiliated Hospital, Zhejiang University School of Medicine, Hangzhou, China.*

^5^ *State Key Laboratory for Diagnosis and Treatment of Infectious Diseases, National Clinical Research Center for Infectious Diseases, Collaborative Innovation Center for Diagnosis and Treatment of Infectious Diseases, The First Affiliated Hospital, Zhejiang University School of Medicine, Hangzhou, China.*

**Supplemental Material**

The strains were submitted by different departments of the hospital, including:

the Department of Neurosurgery (n=157, 16.9%), the Department of General Surgery (n=122, 13.0%), the Department of Respiratory Medicine (n=108, 11.6%), the Intensive Care Unit (n=92, 9.9%), the Critical Care Unit (n=63, 6.8%), the Department of Urological Surgery (n=54, 5.8%), the Department of Neurology (n=51, 5.5%), the Department of Radiotherapy (n=36, 3.9%), the Department of Gastroenterology (n=32, 3.4%), the Department of Hematology (n=26, 2.8%), the Department of Cardiovascular medicine (n=20, 2.1%), the Department of Orthopedics (n=17, 1.8%), the Department of Nephrology (n=15, 1.6%), the Department of Thoracic Surgery (n=15, 1.5%), cardiothoracic surgery department (n=13, 1.3%), the department of rheumatism and immunology (n=10, 1.0%). The number of strains from the departments of rehabilitation, stomatology, interventional medicine, ophthalmology, and endocrinology were in the single digits.

**Supplemental Table S1** Sequences of primers in RT-qPCR

| **Prime** | **Sequence（5’-3’）** |
| --- | --- |
| *AcrA*-F | AACGAACATACCTGGCAACA |
| *AcrA*-R | GTGGAGCTGGTCACTAACGA |
| *AcrB*-F | CGATAACCTGATGTACATGTCC |
| *AcrB*-R | CCGACAACCATCAGGAAGCT |
| *TolC*-F | CTACAAACAGGCGGTGGTCT |
| *TolC*-R | TGTTCAGCTCGTTGATCAGG |
| *rpoB*-F | CTACAAACAGGCGGTGGTCT |
| *rpoB*-R | TGTTCAGCTCGTTGATCAGG |

**Supplemental Figure S1** Variations in carbapenem resistance in *Enterobacter cloacae* complex.

**Supplemental Table S2** Expression of efflux pump-related genes

| **Year** | **Isolates** | **Gene expression** | | | | |
| --- | --- | --- | --- | --- | --- | --- |
|  |  | ***acrA*** | | ***acrB*** | | ***tolC*** |
| / | 700323 | 1 | 1 | | 1 | |
| 2014 | 468 | 0.041±0.014 | 1.289±0.468 | | 2.421±0.667 | |
| 2018 | AR846 | 5.062±0.467 | 6.402±0.225 | | 7.023±1.095 | |
| 2019 | AR1923 | 0.007±0.002 | 1.244±0.217 | | 1.0856±0.366 | |

**Supplemental Table S3** Comparison of OMPs sequences.

| **Year** | **Isolates** | **AmpC** | **OmpC** | **OmpF** | **OmpX** |
| --- | --- | --- | --- | --- | --- |
| 2011 | 482 | MIR-3 | - | - |  |
| 2011 | 611 | ACT-73 | - | - |  |
| 2011 | 617 | ACT-24 |  | - |  |
| 2011 | 719 | ACT-73 | - | - |  |
| 2012 | 354 | LAP-2 | - | - |  |
| 2012 | 545 | ACT-66 | - | - |  |
| 2012 | 951 | ACT-24 | - |  |  |
| 2013 | 157 | ACT-78 |  | - |  |
| 2013 | 265 | ACT-25 |  | - |  |
| 2013 | 594 | ACT-28 | - | - |  |
| 2013 | 915 | ACT-24 |  | - |  |
| 2013 | 953 | ACT-12 |  | - |  |
| 2014 | 303 | ACT-17 | - | - |  |
| 2014 | 468 | ACT-52 |  | - | - |
| 2014 | 852 | ACT-24 |  | - |  |
| 2014 | 940 | ACT-24，MOX-3 |  | - |  |
| 2014 | 1178 | ACT-28 | - | - |  |
| 2015 | 2 | MIR-6 | - | - |  |
| 2015 | 409 | ACT-4 | - |  | - |
| 2015 | 656 | ACT-78 |  | - |  |
| 2015 | 950 | ACT-25 | - | - |  |
| 2015 | 1080 | ACT-25 | - | - |  |
| 2015 | 1082 | ACT-25 | - | - |  |
| 2016 | 1110 | ACT-24，DHA-1 |  | - |  |
| 2017 | 33177 | ACT-24 |  | - |  |
| 2017 | 33344 | ACT-69 |  | - |  |
| 2017 | 33533 | MIR-10 | - | - | - |
| 2017 | 33828 | ACT-45 | - |  |  |
| 2017 | 34597 | ACT-24 |  | - |  |
| 2017 | 35076 | ACT-24 |  | - |  |
| 2017 | 35091 | MIR-3 |  | - |  |
| 2017 | 35140 | ACT-28 |  | - |  |
| 2017 | 36057 | ACT-24 |  | - |  |
| 2017 | 36270 | ACT-45 | - | - |  |
| 2018 | AR304 | ACT-40 | - | - |  |
| 2018 | AR378 | ACT-43 | - |  |  |
| 2018 | AR846 | ACT-28 |  | - |  |
| 2018 | AR940 | ACT-24 | - |  |  |
| 2018 | AR1923 | ACT-40 | - | - |  |
| 2018 | AR1964 | ACT-9 | - | - |  |
| 2019 | AR2034 | ACT-41 |  | - |  |
| 2019 | AR2069 | ACT-24 |  | - |  |
| 2019 | AR2108 | ACT-24 |  | - |  |
| 2019 | AR2259 | ACT-24 | - | - |  |
| 2019 | AR2324 | ACT-24 |  | - |  |
| 2019 | AR2351 | ACT-24 |  | - |  |
| 2019 | AR2379 | ACT-24 |  | - |  |
| 2019 | AR2405 | ACT-24 |  | - |  |
| 2019 | AR2411 | ACT-57 |  | - | - |
| 2019 | AR2612 | ACT-24 |  | - |  |
| 2019 | AR2619 | ACT-24 |  | - |  |
| 2020 | AR2918 | ACT-24 | - | - |  |
| 2020 | AR2928 | ACT-24 | - | - |  |
| 2020 | AR2985 | MIR-3 | - | - | - |
| 2020 | AR3436 | ACT-24 | - |  |  |
| 2020 | AR6514 | MIR-3，LAP-2 | - | - |  |
| 2020 | AR6831 | ACT-55 | - | - | - |
| 2021 | AR7656 | ACT-25 | - | - |  |

The deficiency of OMPs indicated by "-".
